# Therapeutic Plasma Exchange in a 15-kg Child With Atypical Hemolytic Uremic Syndrome: A Case Report

**DOI:** 10.7759/cureus.107418

**Published:** 2026-04-20

**Authors:** Noor Ul Ain, Awais Arshed, Ali Raza, Fatima Raza, Sidra Aman, Musarat Nisa

**Affiliations:** 1 Department of Paediatrics, Combined Military Hospital, Rawalpindi, PAK; 2 Paediatric Nephrology, The Childrens Hospital, Lahore, PAK

**Keywords:** acute kidney failure, anemia and hyperbilirubinemia, clinical remission, massive intravascular hemolysis, therapeutic plasma exchange (tpe)

## Abstract

Atypical hemolytic uremic syndrome (aHUS) is one of the thrombotic microangiopathy (TMA) syndromes characterized by thrombocytopenia, Coombs-negative hemolytic anemia, and renal dysfunction without evidence of Shiga toxin-producing Escherichia coli (E. coli) (STEC) or thrombotic thrombocytopenic purpura (TTP). aHUS is a rare, potentially fatal entity. We present a case of a six-year-old male with vomiting and abdominal pain who developed hematuria and jaundice, along with acute kidney injury (AKI). The patient was diagnosed with aHUS and managed with plasma infusions and exchange, immunosuppression, hemodialysis, and associated kidney-protective measures. He successfully achieved remission and was discharged after a prolonged hospital stay.

## Introduction

Hemolytic uremic syndrome (HUS) is one of the thrombotic microangiopathy (TMA) syndromes characterized by thrombocytopenia, Coombs-negative hemolytic anemia, and renal dysfunction. Atypical HUS (aHUS) is a rare, potentially fatal entity that accounts for approximately 10% of all cases of pediatric HUS [1]. Its incidence ranges from 2.21 to 9.4 cases per million people, with the pediatric age group of zero to four years being most severely affected [2]. aHUS is a complement-mediated disease caused by genetically acquired complement dysregulation, especially in the alternative complement pathway [3]. It is an important cause of pediatric end-stage renal disease and overall morbidity [4].

The diagnosis of aHUS is based on a high index of suspicion and depends on the absence of a preceding Shiga-like toxin-producing Escherichia coli (E. coli) (STEC)-mediated diarrheal disease, with evidence of complement dysregulation in a patient with HUS [5]. This presents a significant challenge in low-income countries where neither advanced diagnostic tools nor the latest treatment options are available. We present a similar case of aHUS from a low-income country where clinical acumen, alongside basic laboratory support and conventional therapeutics, was employed to diagnose and successfully manage pediatric aHUS, achieving remission in a resource-limited setting.

Informed consent was obtained from the parents of the patient before reporting this case.

## Case presentation

A six-year-old male presented to the emergency department (ED) of the pediatric unit with complaints of vomiting and pain in the abdomen for the past seven days. The patient had been in his usual state of health seven days ago when he had started having multiple episodes of non-bilious vomiting. His abdominal pain had started as a dull, generalized ache of 7/10 severity, not associated with food intake and aggravated with episodes of vomiting. There had been associated pallor. He had started passing "cola" colored urine a few hours ago as well. On further inquiry, the parents told the ED doctor that the patient was a thriving child with no preceding history of loose stools, cough, sore throat, rash, or fever. There was no history of a recent trauma. He had no prior history of renal disease or any bleeding disorder and had not reported any urinary complaints before this episode of illness. His family history was negative for any renal or hematologic disorder. He had not been taking any drugs previously. He was immunized and developmentally normal.

On presentation, the patient was a pale, lethargic male with notable facial edema and mild jaundice was found to have a blood pressure of 125/85 mmHg (above 95th centile). His pulse was regular with a rate of 95 beats/minute, he had a temperature of 97 ˚F, a respiratory rate of 18/minute, and oxygen saturation of 96% on room air. His Glasgow Coma Scale (GCS) score was 15/15, and he was fully conscious and responsive. He had a normal chest and precordial examination, and his abdomen was soft and non-tender. He was admitted to the pediatric ICU (PICU), and a workup alongside supportive management was started.

**Figure 1:**
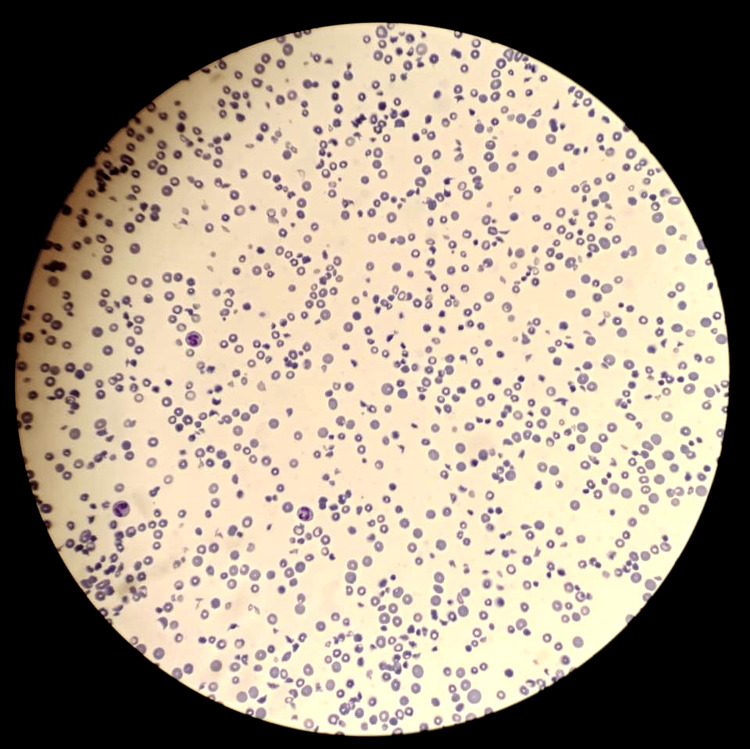
Peripheral blood film of the patient on admission showing abundant schistocytes, indicating severe hemolysis

The initial laboratory studies reported significant anemia with hemoglobin (Hb) of 6.7 g/dL and marked thrombocytopenia of 44,000/μL. The patient's urea level was 51 mmol/L (reference range: 2.8-7.9 mmol/L), and his creatinine was 627 μmol/L (reference range: 34-77 μmol/L). Total bilirubin was slightly raised (40 μmol/L (normal: up to 17μmol/L). Serum lactate dehydrogenase (LDH) was significantly raised, at 5175 (reference range: 150-460 U/L). Blood peripheral film had marked anisopoikilocytosis with polychromasia and a schistocyte count of 60-70/high power field (HPF) (Figure 1). His reticulocyte count was 17.2% with a negative Coombs’ test. Urine microscopy had numerous red blood cells/HPF and a protein of 3+; it was negative for leukocyte esterase and nitrites, and pus cells were 2-3/HPF. His bedside ultrasound abdomen revealed bilateral grade I renal parenchymal changes. His coagulation profile, including prothrombin time (PT) and activated partial thromboplastin time (aPTT), was normal. C-reactive protein (CRP) was 6 mg/L (within normal range).

Thus, the initial impression was hemolytic anemia, associated with thrombocytopenia and acute kidney injury, stage 3 (AKI-III), pointing largely towards the triad related to HUS. Further workup to find any infective, autoimmune, or vasculitic cause was started simultaneously, as presented in Table 1. Initially, urgent dialysis was planned due to the worsening AKI. An 8.5 French size double lumen catheter was inserted bedside by the pediatric intensivist for the patient to undergo hemodialysis, under ultrasound guidance. Red cell concentrates (RCC) were transfused initially to improve Hb, mainly during dialysis sessions. Intravenous (IV) fluids were restricted. Kidney-protective measures, including avoidance of nephrotoxic drugs, were used. He was started on IV empirical antibiotic: ceftriaxone (50 mg/kg/24 hrs). The patient's condition stabilized gradually with the initial three dialysis sessions. 

**Table 1:** Laboratory investigations to ascertain the cause of HUS and rule out other differential diagnoses HUS: hemolytic uremic syndrome

Investigation	Result
Blood culture	No growth
Urine culture	No growth
Stool culture	No growth (negative for E. Coli)
Human immunodeficiency virus (HIV)	Negative
Hepatitis A (IgG/IgM)	Negative
Hepatitis B (HbsAg)	Negative
Hepatitis C (anti-HCV antibody)	Negative
Hepatitis E (IgM)	Negative
Multiplex PCR for respiratory viruses	No virus detected
Anti-double-stranded (ds) DNA antibody	Negative
Antistreptolysin O titers	<100 IU/ml (reference range: <250 IU/mL)
Serum anti-nuclear antibodies (ANA)	Negative
Anti-neutrophil cytoplasmic antibody (ANCA)	Negative
C_3_	0.7↓(reference range: 0.9-1.8 g/L)
C_4_	0.24 (reference range: 0.1-0.4 g/L)

Additional differential diagnoses considered were post-streptococcal glomerulonephritis (PSGN) and thrombotic thrombocytopenic purpura (TTP). PSGN was ruled out due to a lack of supporting history and biochemical evidence of a recent streptococcal throat or skin infection. TTP was deemed less likely because the patient never developed fever or any neurological symptoms during his hospital stay, and TTP is less common than HUS in children; therefore, HUS was the most likely diagnosis. In addition, ADAMTS13 assays were not available in our setting.

IV methylprednisolone (0.8 mg/kg/24hrs) was started by the pediatric nephrologist after reviewing all the evidence of ongoing hemolysis, and additionally, IV omeprazole (10 mg/kg/24 hrs) was started alongside. Mycophenolate mofetil (MMF) (1200 mg/m^2^/24 hrs x twice daily) in syrup form was started. Tab amlodipine 2.5 mg x once daily was advised for controlling blood pressure. Initial urine output (UO) was 1.6-2 ml/kg/hr. However, the patient's hematuria and drop in Hb secondary to ongoing hemolysis persisted. This was further managed with infusions of fresh frozen plasma (FFP) at a dose of 10mg/kg/dose twice a day, anticipating a complement cascade-driven etiology, as all the possible infectious and autoimmune workup returned negative. Platelets, however, were not transfused, expecting deterioration of clinical and hematologic parameters. Intermittent episodes of high blood pressure were managed with hydralazine injection.

A fresh peripheral blood film revealed persistent levels of schistocytes (30-40/HPF), and the complete blood picture showed a steady drop in Hb despite intermittent RCC transfusions and persistent thrombocytopenia. Hematuria also continued. Following a hematology consultation during a multidisciplinary team meeting, therapeutic plasma exchange (TPE) sessions were planned for the patient. TPE in a child weighing 15 kg had not been previously performed at our center, owing to the increased risk of hemodynamic instability. After briefing the parents in detail and obtaining informed consent, the patient underwent a total of eight sessions of TPE, and his condition improved drastically.

The risk of hemodynamic instability was minimized by using RCC to prime the extracorporeal volume of the plasmapheresis machine circuit, maintaining slow exchange rates and careful blood pressure monitoring. The patient had a taxing inpatient stay with dialysis requirements every other day or after two to three days, as the AKI only improved gradually and renal function tests remained fairly high. However, the AKI after running a refractory course did show improvement. His hematuria settled gradually, reflected in the decreasing number of schistocytes on the peripheral blood films done serially, and Hb stabilized with a lesser need to transfuse. He had undergone a total of eight sessions of TPE and five sessions of HD to achieve remission.

His C3 levels were mildly reduced, with normal C4. Detailed complement studies were not available in our setting. The patient was offered genetic studies after no infectious cause was identified for HUS, but due to affordability issues, these were not performed. Thus, given his age of >5 years, the absence of a possible infectious etiology of HUS, the lack of a preceding history of diarrheal illness, and a refractory clinical course requiring immunosuppression, hemodialysis, and plasma infusions and exchange, a presumptive diagnosis of atypical HUS was made.

Methylprednisolone injections were discontinued and replaced with oral prednisolone (initially at 1 mg/kg/24 hours), with a plan to taper later. The patient was discharged after a month of inpatient stay while continuing immunosuppressive therapy with MMF and intermittent FFP administration (twice weekly initially), with close follow-up by a pediatric nephrologist. Tablet amlodipine was continued for his hypertension. At the next fortnightly follow-up, the patient remained in remission, with a schistocyte count of 1-2/HPF and a reticulocyte count of 0.5%, maintaining his Hb and platelet counts of 12.8 g/dL and 281,000/μL, respectively.

## Discussion

aHUS is a consequence of genetic mutations in the alternative complement pathway that result in uninhibited complement activation and the resultant TMA spectrum disease. The prognosis of this disease remains guarded without aggressive management and therapeutics, with mortality of up to 25% in patients experiencing a first attack, and about 50% of patients suffer from progressive renal disease and eventually become dialysis-dependent [4].

Our patient's diagnosis of aHUS was in line with the diagnostic criteria laid down by the Joint Committee of the Japanese Society of Nephrology and the Japanese Pediatric Society [5]. An accurate diagnosis is established by evidence of anemia (Hb <10 g/dL) due to hemolysis, manifested by elevated LDH levels, reduced haptoglobin levels, and the presence of red cell fragments with schistocytes on a peripheral blood film. This is coupled with thrombocytopenia (platelets <150,000/μL) and renal dysfunction (creatinine levels 1.5 times or greater than baseline for the specific pediatric age group). STEC-HUS and TTP must be ruled out. The main diagnostic challenge in our country was the limited availability of diagnostic laboratory panels and the inability of the patient to afford extensive complement assay testing and genetic testing. Therefore, we relied on available resources and successfully managed the patient.

Plasma therapy, including plasma infusions and exchange, is considered the mainstay of treatment for aHUS, and it helps keep the disease under control. The main challenge in our case was performing TPE in a 15-kg patient, with a significant risk of hemodynamic instability, which was overcome using meticulous priming of the circuit and the use of RCC transfusions during the procedure to avoid hypoxia and hypotension. Currently, targeted treatment is available, namely a recombinant monoclonal antibody against the complement C5 protein (eculizumab). This drug has been proven to be very effective in maintaining aHUS in remission with good renal recovery [6]. However, difficult-to-treat relapses of aHUS have been reported both with plasma therapy and after discontinuation of complement-blocking therapy. Our patient was treated not only with plasma therapy but also with an improvised immunosuppression regimen consisting of steroids and MMF due to the unavailability of eculizumab.

Pediatric aHUS has a refractory and relapsing course and is often fatal, especially in the infantile age group. A case of a six-month-old infant having anti-factor H antibody-mediated HUS resulted in fatality following multi-organ failure [7]. Regarding disease outcomes, a National Surveillance study conducted in Australia followed 14 patients with aHUS and reported dialysis requirement on initial presentation, a relapsing course, especially in the first 12 months of disease occurrence, end-stage renal disease, dialysis dependency, and need for kidney transplantation and further graft loss, showing the devastating course of this disease [8].

## Conclusions

aHUS is a consequence of genetic mutations in the alternative complement pathway that result in uninhibited complement activation and a resultant TMA spectrum disorder. The diagnosis of aHUS is based on a high index of suspicion and depends on the absence of a preceding STEC-mediated diarrheal disease, with evidence of complement dysregulation in a patient with HUS. The disease runs a refractory, relapsing course in children and requires meticulous management strategies to achieve remission, including immunosuppression, plasma infusions and exchange, and kidney-protective measures.
